# The variable phenotype and low-risk nature of *RAS*-positive thyroid nodules

**DOI:** 10.1186/s12916-015-0419-z

**Published:** 2015-08-07

**Authors:** Marco Medici, Norra Kwong, Trevor E. Angell, Ellen Marqusee, Matthew I. Kim, Mary C. Frates, Carol B. Benson, Edmund S. Cibas, Justine A. Barletta, Jeffrey F. Krane, Daniel T. Ruan, Nancy L. Cho, Atul A. Gawande, Francis D. Moore, Erik K. Alexander

**Affiliations:** Thyroid Section, Division of Endocrinology, Hypertension and Diabetes, The Brigham and Women’s Hospital and Harvard Medical School, 75 Francis Street, PBB-B4 Room 417, Boston, MA 02115 USA; Department of Pathology, The Brigham and Women’s Hospital and Harvard Medical School, Boston, MA USA; Department of Radiology, The Brigham and Women’s Hospital and Harvard Medical School, Boston, MA USA; Department of Surgery, The Brigham and Women’s Hospital and Harvard Medical School, Boston, MA USA

## Abstract

**Background:**

Oncogenic mutations are common in thyroid cancers. While the frequently detected *RAS*-oncogene mutations have been studied for diagnostic use in cytologically indeterminate thyroid nodules, no investigation has studied such mutations in an unselected population of thyroid nodules. No long-term study of *RAS*-positive thyroid nodules has been performed.

**Methods:**

We performed a prospective, blinded cohort study in 362 consecutive patients presenting with clinically relevant (>1 cm) thyroid nodules. Fine needle aspiration cytology and mutational testing were obtained for all nodules. Post-operative histopathology was obtained for malignant or indeterminate nodules, and benign nodules were sonographically followed. Histopathological features were compared between *RAS*- and *BRAF*-positive malignancies. *RAS*-positive benign nodules were analyzed for growth or cellular change from prior aspirations.

**Results:**

Overall, 17 of 362 nodules were *RAS*-positive. Nine separate nodules were *BRAF*-positive, of which eight underwent surgery and all proved malignant (100 %). Out of the 17 *RAS*-positive nodules, ten underwent surgery, of which eight proved malignant (47 %). All *RAS*-positive malignancies were low risk – all follicular variants of papillary carcinoma, without extrathyroidal extension, metastases, or lymphovascular invasion. *RAS*-positivity was associated with malignancy in younger patients (*P =* 0.028). Of the nine *RAS*-positive benign nodules, five had long-term prospective sonographic follow-up (mean 8.3 years) showing no growth or signs of malignancy. Four of these nodules also had previous aspirations (mean 5.8 years prior), all with similar benign results.

**Conclusions:**

While *RAS*-oncogene mutations increase malignancy risk, these data demonstrate a low-risk phenotype for most *RAS*-positive cancers. Furthermore, cytologically benign, yet *RAS*-positive nodules behave in an indolent fashion over years. *RAS*-positivity alone should therefore not dictate clinical decisions.

## Background

Over the last two decades, the discovery of molecular pathways critical to oncogenic transformation has dramatically altered our understanding of thyroid malignancy. Reports initially suggested that nearly 70 % of thyroid cancers harbor single gene mutations in the BRAF or RAS pathways, or balanced translocations of *RET/PTC*, or *PAX8/PPARγ* [1, 2]. More recently, reports confirm an oncogenic mutation in 97 % of well-differentiated papillary carcinomas [3]. Such mutations can increasingly be identified in both preoperative fine needle aspiration (FNA) cytology specimens and on post-operative histopathology. Many believe that the synergistic use of microscopic and molecular analysis is destined to improve the clinical management of this illness [4].

Mutations or translocations identified in most thyroid carcinomas are known to activate pathways regulating cellular growth, development, and/or malignant transformation, which has led to the assumption that thyroid nodules harboring such mutations are either cancerous, or at high risk for eventual malignant transformation [5–7]. However, observational data question this assumption as absolute and suggest that all such pathways or activating mutations may not prove equally oncogenic or may require secondary molecular ‘hits’ before behaving in a malignant fashion. In particular, clinical outcomes of patients with mutations in the N-, K-, or H- isoforms of the *RAS* gene are highly variable [7]. In contrast to homogeneous *BRAF* V600E mutations which strongly associate with higher-risk papillary carcinoma, initial observations suggest *RAS* mutations associate with variable histology, ranging from benign disease, to low-risk malignancy, to anaplastic carcinoma [7–10].

However, few prospective blinded assessments of *RAS*-positive thyroid nodules (treated or untreated) have been performed. Such an assessment is critically important, as diagnostic testing for these mutations is now widely available. To many clinicians, identification of an activating *RAS* mutation may prompt a belief that malignancy has been identified [7]. This leads to downstream clinical recommendations usually favoring surgical resection. Such treatment is highly beneficial in many scenarios, but for those with benign disease, has subjected the patient to unnecessary cost, operative morbidity, and significant risk [11, 12]. Past experience involving papillary microcarcinoma provides a cautionary parallel supporting the importance of further study of *RAS*-positive thyroid nodules. For decades, widespread belief that all papillary carcinoma posed danger prompted recommendations for active removal of such thyroid nodules [13, 14]. This was true even when nodules were smaller than 1 cm and ultrasound confirmed the absence of abnormal adenopathy. Prospective data debunked this belief, confirming the indolent nature of these papillary microcarcinomas [15]. Expert guidelines now recommend against evaluation of most nodules <1 cm, and/or surgical treatment of sub-centimeter papillary carcinomas [16, 17].

We performed a prospective study of clinically relevant thyroid nodules, including ultrasound-guided FNA and gene mutation analysis. Blinded mutational analysis and histopathologic interpretation was performed. Our goal was not to validate the performance of a molecular diagnostic test on cytologically indeterminate nodules, as such investigations have previously been performed. Rather, we uniquely sought to evaluate the molecular profiles of a large consecutive cohort of thyroid nodules >1 cm presenting for FNA. By doing so, we sought to understand the molecular profiles detected in an unselected nodule population whether cytologically benign, indeterminate, or malignant. Furthermore, as a large proportion of our patients also participate in an on-going prospective, long-term cohort study assessing the natural history of thyroid nodules, we hypothesized that several mutation-positive nodules would also have long-term sonographic follow-up for analysis. If so, assessment for potential malignant transformation – in particular when observing the natural history of *RAS*-positive thyroid nodules – would be for the first time possible.

## Methods

We performed a prospective, blinded study of euthyroid patients seeking care of thyroid nodules >1 cm in diameter. Patients were referred to the thyroid biopsy clinic at the Brigham and Women’s Hospital (Boston, MA, USA) between July 2010 and October 2012, and evaluated according to current clinical practice guidelines [16]. Ultrasound evaluation was performed by one of four radiologists with expertise in thyroid evaluation, using a 6–15 mHz transducer. Following informed consent, ultrasound-guided FNA was performed by one of three thyroidologists. Three needle passes from the same nodule using 25 g needles were rinsed into a liquid-based solution (CytoLyt®; Cytyc Corp., Marlborough, MA, USA), constituting a single aspiration. An additional sample was then obtained and shipped to a centralized CLIA-certified laboratory at Asuragen, Inc. (Austin, TX, USA) where mutational analysis was performed as part of the miR*Inform* Thyroid® diagnostic test [18]. As previously described, this test evaluates 17 distinct genetic alterations, including 14 *BRAF*, *K-*, *N-*, or *H-RAS* mutations, and three *PAX8-PPARγ* and *RET-PTC* rearrangements [18]*.* Of 391 nodules enrolled for initial evaluation, 11 yielded insufficient nucleic acid for mutational testing. A separate consecutive group of 15 separate nodules all showed an uncharacteristic molecular result (double positive for *PAX8-PPARG* and *RAS*) suggesting external cross-contamination during processing of this series, and were therefore excluded. Three nodules were excluded as a result of a protocol deviation (one delayed shipping) or study dropout (n = 2). This resulted in a final population of 362 nodules from 318 patients.

FNA cytology was classified according to the Bethesda system for reporting thyroid cytopathology [19]. Benign cytologic results most often prompted a conservative, non-operative recommendation. Cytologically indeterminate and malignant nodules most often prompted a recommendation for surgical resection. Midway through this study, Afirma gene expression classifier (GEC) testing became available, and was applied to a minority of low-risk patients with initial atypia of undetermined significance (AUS) cytology. If Afirma testing was benign, nodules were treated similarly to those with benign cytology [20]. Following thyroidectomy, histopathology interpretation was performed, which frequently involved multi-expert review and consensus. All histopathologic interpretations were blinded to molecular results. Similarly, molecular interpretation was performed without knowledge of any clinical or pathologic findings. At study completion, results were combined and interpreted.

The Brigham and Women’s Hospital thyroid nodule clinic has prospectively enrolled all patients evaluated between 1995–present in an ongoing clinical trial assessing the natural history of thyroid nodules [21]. We analyzed all past and present thyroid sonographic imaging in patients with *RAS*-positive, cytologically benign thyroid nodules. For some of the patients, a separate ultrasound-guided FNA of the target nodule had also been performed at an earlier time point before enrollment in the current study. This was usually at a separate facility or by a separate provider.

For the purposes of this study, we sought to specifically describe the histologic outcome of all thyroid nodules positive for *N-*, *H-*, and *K-RAS* mutations, as these mutations are common yet observations suggest a variable phenotype. For *RAS*-positive nodules confirmed malignant by histopathology, we documented the cancer type and specific papillary thyroid carcinoma (PTC) variant. Nodule size, as well as multifocality, lymphovascular invasion, extrathyroidal extension, and local metastatic adenopathy were similarly documented. For *RAS*-positive nodules with benign cytology, we reviewed all available sonographic and cytologic reports documenting thyroid nodule size, abnormal adenopathy, or other signs of malignant behavior, with a follow-up time of at least 6 months. Growth was defined as a >20 % change in the largest two nodule dimensions.

This protocol was approved by the Investigational Review Board of the Brigham and Women’s Hospital, and all patients provided written informed consent for participation and publication of individual patient data, including the data described in Tables 2 and 4. No patients received a stipend for participating in this study. ANOVA was used to compare the mean age between patients with *RAS*-positive benign and malignant nodules. Statistical analysis was performed using SPSS version 22 (SPSS IBM, Armonk, NY, USA), and a *P* value <0.05 was considered significant.

## Results

We prospectively enrolled 318 patients with 362 clinically relevant thyroid nodules (>1 cm), whose baseline characteristics are shown in Table 1. Following blinded molecular analysis, 17 nodules were positive for mutations in the *K-*, *N-*, or *H- RAS* genes, while nine separate nodules harbored V600E *BRAF* mutations. Three additional nodules were positive for translocations involving the *PAX8-PPARγ* genes, while no *RET-PTC* translocations were found. Ultimately, 33 of 362 (9.1 %) nodules proved malignant following surgical resection and blinded histopathologic assessment, including eight *BRAF*-positive and eight *RAS*-positive nodules. Of the 17 *RAS*-positive nodules, ten were referred to surgery because of abnormal or malignant cytology, while seven did not have surgery as their biopsy was benign (six cytologically ‘benign’; one nodule cytologically AUS but subsequent ‘benign’ GEC). Of the ten *RAS*-positive nodules referred to surgery, eight proved histologically malignant, while two were histologically benign. In summary, 8 of 17 *RAS* mutation-positive nodules (47 %) were malignant by microscopic analysis, while the remainder were benign.

**Table 1:** Baseline characteristics of the study population and the *RAS*-positive subgroup

	Number of patients	Number of nodules	Female (%)	Age (years)	Nodule size (cm)	Proportion malignant^a^	Mutations detected
Total population	318	362	78.7 %	Range: 21.8–87.7 Median: 55.0	Range: 1.0–6.6 Median: 1.9	33 nodules^b^ (9.1 %)	17 RAS + 9 BRAF + 3 PAX8-PPARγ +^c^
*RAS*-positive nodules	17	17	88.2 %	Range: 27.1–63.5 Median: 46.0	Range: 1.0–5.6 Median: 1.8	8 nodules (47.1 %)^d^	8 HRAS+ (3 G12V, 2 Q61K, 2 Q61R, 1 G13R) 7 NRAS+ (6 Q61R, 1 Q61K) 2 KRAS+ (1 G12V)

^a^Histologically proven; ^b^of the 362 nodules, 63 were referred to surgery because of non-benign cytology, of which 33 proved malignant; ^c^of the three PAX8-PPARγ-positive nodules, two underwent surgery and were proven benign. The third nodule underwent Afirma GEC testing, which was also benign; ^d^of the 17 *RAS*-positive nodules, ten were referred to surgery because of indeterminate or malignant cytology, of which eight proved malignant. GEC, gene expression classifier

### *RAS*-positive thyroid malignancies

Table 2 shows the characteristics of the *RAS*-positive thyroid malignancies. All (n = 8) were follicular variants of PTC (fvPTC), and all demonstrated very low-risk characteristics. Specifically, there was no evidence of lymphovascular invasion, extrathyroidal extension, local lymph node metastases, or distant metastases in any *RAS*-positive thyroid cancers. Furthermore, all were encapsulated, or partially encapsulated/well-circumscribed malignancies, and there were no disease-specific deaths. For comparison, we evaluated the histologic characteristics of the *BRAF*-positive thyroid malignancies. Compared to the *RAS*-positive malignancies, the *BRAF*-positive malignancies were equal in size (2.1 cm). However, they had less favorable histological characteristics as shown in Table 3. These data suggest a more indolent phenotype among many *RAS*-positive thyroid cancers.

**Table 2:** Characteristics of *RAS*-positive thyroid malignancies

Subject number	Sex	Age (years)	*RAS* mutation	Nodule size (mm) and parenchyma	FNA result	Histopathology	Encapsulated	Extrathyroidal extension	Lymph node metastases	Distant metastases
1	Female	27	HRAS G12V	10 × 7 × 4 Solid No calcifications	Follicular neoplasm	PTC multifocal, 1.2 cm	Partially-encapsulated/well-circumscribed	No	No	No
2	Female	46	HRAS Q61R	14 × 9 × 9 Solid No calcifications	Suspicious for papillary carcinoma	PTC follicular variant, 1.1 cm	Partially-encapsulated/well-circumscribed	No	No	No
3	Male	61	HRAS Q61R	36 × 23 × 21 25–50 % Cystic No calcifications	Suspicious for papillary carcinoma	PTC follicular variant, 2.6 cm	Partially-encapsulated/well-circumscribed	No	No	No
4	Female	33	HRAS G13R	20 × 18 × 16 Solid No calcifications	Suspicious for papillary carcinoma	PTC follicular variant, 1.8 cm	Encapsulated	No	No	No
5	Female	44	NRAS Q61R	22 × 11 × 10 Solid No calcifications	Malignant – papillary carcinoma	PTC follicular variant, 1.1 cm	Partially-encapsulated/well-circumscribed	No	No	No
6	Female	45	NRAS Q61R	18 × 14 × 7 Solid No calcifications	Malignant – papillary carcinoma	PTC follicular variant, 1.0 cm	Encapsulated	No	No	No
7	Female	44	NRAS Q61R	18 × 14 × 12 Solid No calcifications	Follicular neoplasm	PTC follicular variant, 1.6 cm	Encapsulated	No	No	No
8	Female	33	NRAS Q61R	31 × 23 × 18 Solid No calcifications	Atypia of undetermined significance	PTC follicular variant, 3.1 cm	Encapsulated^a^	No	No	No

^a^One focus of potential capsular penetration. FNA, fine needle aspiration; PTC, papillary thyroid carcinoma

**Table 3:** Comparison of *RAS*-positive and *BRAF*-positive papillary thyroid cancers

Mutation	Positive predictive value (test specificity)	Tumor size (cm)	Histological subtype	Lymphovascular invasion	Extrathyroidal extension	Lymph node metastases
*RAS*-positive cancers (n = 8)	47 % (97.3 %)	Range: 1.0–3.6 Median: 1.9	8 – follicular variant PTC	0/8 (0 %)	0/8 (0 %)	0/8 (0 %)
*BRAF*-positive cancers (n = 8^a^)	100 % (100 %)	Range: 1.0–4.8 Median: 1.4	6 – classical variant PTC^b^ 1 – tall cell variant PTC 1 – follicular variant PTC	5/8 (62.5 %)	1/8 (12.5 %)	1/8 (12.5 %)

^a^Nine *BRAF* mutations were detected in the cohort. However, one patient did not pursue surgery because of other medical conditions. Therefore eight *BRAF*-positive cases are shown^; b^including two classical variant PTCs with tall cell features. PTC, papillary thyroid carcinoma

### *RAS*-positive benign thyroid nodules

Nine *RAS*-positive thyroid nodules proved benign (two histologically benign; six cytologically benign; one benign GEC). Characteristics of the *RAS*-positive benign nodules are shown in Table 4. These nodules averaged 2.1 cm in largest dimension at study entry. While four nodules had not been previously evaluated in our thyroid nodule clinic, five nodules had undergone previous sonographic imaging and evaluation, including four with prior ultrasound-guided FNA. We therefore analyzed the repeated sonographic assessment of five *RAS*-positive cytologically benign nodules over time. No significant nodule growth was confirmed in all five (100 %) cases over a mean duration of 8.3 years (range: 3.1–24.0 years). Furthermore, no abnormal adenopathy or other sonographically worrisome features were identified throughout follow-up in all patients. For comparison, we randomly selected age and sex matched mutation-negative controls at a 3:1 ratio (i.e., 15 controls) and analyzed the same parameters. Over a comparable mean follow-up time of 7.5 years (range: 2.8–24.0 years), an average 4.6 mm increase in largest dimension was observed from these cytologically benign, mutation-null nodules.

**Table 4:** Characteristics and sonographic follow-up of *RAS*-positive benign nodules

Subject number	Sex	Age at study entry (years)	*RAS* mutation	Nodule size (mm) and parenchyma at study entry	FNA cytology at study entry	Previous or subsequent ultrasound	Duration of sonographic follow-up	Previous FNA cytology
						(date, size (mm), parenchyma)		(date, result)
9	Female	52	HRAS G12V	11/2010, 18 × 5 × 11, <25 % Cystic	Benign	02/2013, 16 × 11 × 9, Cystic (<25 %)	3.9 years (no growth)	Not performed
						10/2014, 17 × 10 × 8, N/A		
10	Female	52	HRAS Q61K	03/2012, 16 × 14 × 9, Solid	Benign	09/2008, 17 × 12 × 11, Solid	3.4 years (no growth)	11/2008, Benign cytology
						11/2008, 17 × 10 × 10, N/A		
						01/2012, 16 × 15 × 9, Solid		
11	Female	37	KRAS G12V	09/2011, 15 × 11 × 9, <25 % Cystic	Benign	-	N/A	Not performed
12	Female	54	KRAS G12V	10/2010, 14 × 14 × 8, Solid	Benign	07/1990, 13 × 12 × 7, N/A 10/1993, 14 × 12 × 8, N/A 05/1998, 15 × 13 × 8, Solid 06/1998, 15 × 13 × 8, Solid 10/1999, 15 × 13 × 7, Solid 07/2003, 15 × 14 × 7, Solid 07/2005, 14 × 14 × 8, Solid 10/2007, 15 × 14 × 7, Solid 01/2008, 13 × 12 × 7, Solid 08/2009, 14 × 13 × 8, Solid 09/2010, 16 × 15 × 9, Solid 07/2011, 16 × 13 × 7, Solid 01/2012, 15 × 15 × 7, Solid 01/2013, 16 × 15 × 8, Solid 07/2014, 15 × 14 × 9, Solid	24.0 years (no growth)	06/1998, Benign cytology
13	Female	54	NRAS Q61K	04/2011, 15 × 12 × 11, <25 % Cystic	Benign	05/2006, 15 × 9 × 9, Cystic (<25 %) 09/2006, 14 × 10 × 9, Cystic (<25 %) 06/2009, 12 × 11 × 11, Cystic (<25 %) 04/2011, 16 × 13 × 10, N/A 04/2012, 17 × 12 × 12, Cystic (<25 %) 06/2013, 15 × 12 × 10, Cystic (<25 %)	7.0 years (no growth)	09/2006, Benign cytology
14	Female	30	NRAS Q61R	09/2011, 16 × 14 × 9, Solid	Benign	08/2008, 13 × 13 × 8, Solid 12/2008, 13 × 13 × 9, Solid 07/2011, 15 × 14 × 8, N/A	3.1 years (no growth)	12/2008, Benign cytology
15	Female	62	NRAS Q61R	01/2012, 17 × 14 × 12, Solid	AUS + Afirma GEC ‘Benign’	N/A	N/A	04/2012, AUS
								05/2012, Afirma GEC: ‘benign’
16	Female	64	HRAS G12V	11/2010, 23 × 19 × 15, <25 % Cystic	AUS	N/A	N/A	01/2011, Surgery^a^: benign histology
17	Male	46	HRAS Q61K	03/2012, 56 × 45 × 33, Solid	Follicular neoplasm	N/A	N/A	05/2012, Surgery^a^: benign histology

^a^Surgery after study entry. AUS, atypia of undetermined significance; FNA, fine needle aspiration; GEC, gene expression classifier; N/A, not available

Four of six *RAS*-positive, cytologically benign nodules had undergone a separate secondary biopsy at a time point preceding entry into this current study. All four previous aspirates (100 %) were cytologically benign when performed at mean 5.8 years prior to study entry, confirming no change in cellular morphology to the present. One other nodule demonstrated AUS at cytology on study entry, but was benign on subsequent GEC testing. This nodule was therefore considered benign. Finally, we compared the age of patients with *RAS*-positive thyroid nodules which proved malignant to those which proved benign. Patients with *RAS*-positive benign nodules were on average 13 years older (55.1 (3.7) (mean (SD)) versus 41.9 (4.0) years; *P* = 0.028) than patients with *RAS*-positive malignant nodules.

## Discussion

Over the last decade, our understanding of the molecular pathways underpinning thyroid cancer has dramatically increased. This has improved care, though simultaneously fostering many clinical assumptions influenced by the population being studied. Nearly all investigations of molecular mutations have been performed on cancerous or cytologically indeterminate thyroid nodules. Importantly, this was not the goal of our study. We sought to present the first blinded, prospective analysis of *RAS* mutations in a general cohort of patients presenting with nodular disease, to better understand the meaning of such mutations and the natural history of *RAS*-positive nodules. Our data demonstrate a variable and generally low-risk phenotype among most *RAS*-positive nodules. *RAS*-positive status predicts thyroid cancer in 47 % of cases, though is also associated with a large proportion of thyroid nodules with benign histology, benign cytology, and indolent clinical characteristics during long-term conservative follow-up.

In our population, *RAS*-positive thyroid cancers were uniformly low risk – all histologically confirmed to be fvPTC and encapsulated, and all without lymphovascular invasion, extrathyroidal extension, or local lymph node metastases. While many have argued that *RAS*-positive nodules are destined to behave in a malignant fashion, our data suggest this assumption should be viewed with caution. Several of the *RAS*-positive, cytologically benign thyroid nodules in our study cohort had previously been aspirated at an average of 5.8 years prior. This confirms the lack of meaningful cellular transformation over time. Furthermore, highly accurate sonographic assessment of these nodules during a mean 8-year follow-up (and up to 24 years in one patient) confirmed no growth concerning for malignant transformation. Together, these data suggest a far more indolent phenotype inherent to many *RAS*-positive thyroid nodules than previously described.

In the current study, all *RAS*-positive malignancies were fvPTCs (Table 3), while there were no *RAS* mutations detected in classical variant PTCs. This is interesting in the light of the results of the study of Castro et al., who showed that fvPTCs are molecularly more similar to follicular carcinoma than to classical variant PTCs [22]. This included a higher percentage of *RAS* mutations in fvPTCs than generally reported in classical variant PTCs.

The ability of activated mutant *RAS* to induce thyroid neoplasia *in vitro* has been well established [23–25]. Furthermore, the cellular pathways stimulated by mutated *RAS*, including the MAPK and PI3/AKT signaling pathways, have been linked to thyroid tumorigenesis [23, 26]. However, the *RAS* gene encodes a family of three isoforms, NRAS, HRAS, and KRAS, with numerous different mutations described. Some studies have demonstrated a tight association between mutated *RAS* and follicular thyroid carcinoma, while others confirm a high prevalence of *RAS* mutations in benign adenomas or fvPTC [7]. These findings confirm that the MAPK and PI3/AKT cellular pathways are intimately involved in cellular growth and differentiation, but also demonstrate that numerous genetic and epigenetic factors likely contribute to the clinical phenotype. This is exemplified by recent analysis of the thyroid cancer genome atlas [3]. Our data support such findings, while broadening our understanding specific to *RAS*-positivity in a large population, inclusive of mostly benign nodules.

Other published data support our findings. It is again important to note that most investigations have studied *RAS* mutations only in populations with malignant or indeterminate cytology [2, 27–29]. Similar to us, however, Moses and colleagues performed a blinded and prospective mutational analysis of 417 patients presenting with nodular disease [9]. In this cohort, 21 *RAS*-positive nodules were identified (4.6 % prevalence), very similar to the 4.7 % detected in our population. While 12 *RAS* mutations were identified among their 194 patients with abnormal cytology, nine additional *RAS* mutations were identified in the remaining 257 benign nodules. In total, 6 of 21 (29 %) *RAS*-positive nodules proved histologically malignant. This rate is comparable to our finding of 47 %. Unlike our investigation, however, no previous sonographic analysis or FNA were performed. Nonetheless, these data independently support our conclusion that *RAS* mutations are commonly detected in benign nodules.

While the number of samples in the current study is too limited from which to draw firm conclusions regarding the distribution of *RAS* mutation subtypes in benign or malignant nodules, it is noteworthy that two *KRAS* mutations were detected in benign nodules, while none were detected in malignant nodules. Interestingly, Radkay and colleagues have also studied 204 FNA cases with *RAS* mutations (mostly indeterminate cytology) with corresponding surgical resection pathological specimens, similarly demonstrating that mutations in *KRAS* were associated with a significantly lower risk of carcinoma (41.7 %) compared to nodules with *HRAS* (95.5 %) and *NRAS* (86.8 %) mutations [29].

It is unclear why *RAS*-positive thyroid nodules behave in a less virulent manner, though several hypotheses can be considered. One possibility is that the different *RAS* mutations affect downstream protein function to variable degrees. This hypothesis is supported by parallel evidence observed in medullary thyroid carcinoma patients with activating *RET* mutations, in which over 50 unique mutations are described [30]. These data confirm that different genetic mutations in the same oncogene lead to variable malignant risk. Separately, it is increasingly likely that a two-hit hypothesis is necessary for malignant transformation in many *RAS*-positive nodules, especially those associated with aggressive disease [31]. Further investigation is required to better address these hypotheses.

Importantly, we do not advocate performing *RAS*-mutational testing on all thyroid nodules at time of presentation for several reasons. First, FNA cytology proved more accurate than *RAS*-analysis, as all thyroid malignancies were identified in nodules with indeterminate or malignant cytology. Second, such an approach has not been shown to be cost-effective nor to produce an improved health outcome. We realize, however, that some may nonetheless argue that detection of *RAS*-mutational status is important beyond simply its use in cytologically indeterminate FNAs. *RAS*-positive nodules indeed carry a higher risk for malignancy compared to *RAS*-negative nodules, and *RAS*-positive status may also affect prognostic and therapeutic decisions once malignant [8].

Our data do lend preliminary support for surgical removal of *RAS*-positive nodules in younger individuals with non-benign cytology, but question this approach in *RAS*-positive benign nodules. The majority of our study cohort with cytologically benign, yet *RAS*-positive nodules have been closely followed by clinical and sonographic assessment. During a follow-up that ranged from 3–24 years, no significant growth or malignant transformation was documented. Furthermore, repeat aspiration confirmed the presence of consistently benign cytology. Thus, any recommendation for thyroidectomy must balance the presumed benefits of such a procedure against its known risks [11, 12].

We acknowledge limitations to our study. Notably, we recruited patients from a single institution. However, the fact that our clinic evaluates >95 % of all patients seeking thyroid nodule care in our healthcare system improves generalizability of these results and limits selection bias. We similarly acknowledge the lack of universal long-term follow up of all *RAS*-positive nodules. Though our data depict no growth via highly accurate sonographic follow-up of five such patients, it is possible such benign nodules would indeed transform into malignant processes over enough time. Importantly, however, our study also confirmed an impressive low-risk histologic profile to most *RAS*-positive malignancies. Thus, if an observational strategy was followed, repeat sonographic follow-up or repeat FNA may allow the patient and physician to arguably detect any future malignancy while still at a treatable stage. Further prospective study of this hypothesis is required. Finally, one may argue that our study should have been restricted to nodules with indeterminate cytology as this population is in whom mutational testing is currently recommended. However, that was not the goal of our study as we sought to investigate *RAS*-status in all nodules regardless of cytology results. We believe this approach provided us the unique opportunity to investigate the natural history and malignant risk of clinically relevant *RAS*-positive nodules in an unbiased fashion.

## Conclusions

In summary, our data depict the blinded, prospective evaluation of *RAS*-mutational status in a population of patients presenting with clinically relevant thyroid nodules, and demonstrate a more indolent and variable phenotype than previously described. *RAS*-positive thyroid nodules, especially in older individuals, frequently demonstrate a benign phenotype. These data therefore support the utility of FNA cytology in guiding the clinical management of *RAS*-positive nodules. Cytologically benign nodules, even if *RAS*-positive, may be candidates for a non-operative observational strategy of repeated sonographic evaluation or FNA. Even if malignant, most *RAS*-positive thyroid nodules appear to be low-risk histologically, and thus are likely to be highly treatable.

## Abbreviations

ANOVA: Analysis of variance
AUS: Atypia of undetermined significance
CLIA: Clinical Laboratory Improvement Amendments
FNA: Fine needle aspiration
fvPTC: Follicular variant of papillary thyroid carcinoma
GEC: Gene expression classifier
PTC: Papillary thyroid carcinoma
